# Electrochemical properties of titanium nitride nerve stimulation electrodes: an *in vitro* and *in vivo* study

**DOI:** 10.3389/fnins.2015.00268

**Published:** 2015-08-04

**Authors:** Suzan Meijs, Morten Fjorback, Carina Jensen, Søren Sørensen, Kristian Rechendorff, Nico J. M. Rijkhoff

**Affiliations:** ^1^SMI, Department of Health, Science and Technology, Aalborg UniversityAalborg, Denmark; ^2^Neurodan A/SAalborg, Denmark; ^3^Danish Technological InstituteÅrhus, Denmark

**Keywords:** implantable neural prosthesis, foreign body reaction, electrical stimulation, electrochemical impedance spectroscopy, voltage transient measurements

## Abstract

The *in vivo* electrochemical behavior of titanium nitride (TiN) nerve stimulation electrodes was compared to their *in vitro* behavior for a period of 90 days. Ten electrodes were implanted in two Göttingen minipigs. Four of these were used for electrical stimulation and electrochemical measurements. Five electrodes were kept in Ringer's solution at 37.5°C, of which four were used for electrical stimulation and electrochemical measurements. The voltage transients measured *in vivo* were 13 times greater than *in vitro* at implantation and they continued to increase with time. The electrochemical properties *in vivo* and the tissue resistance (R_tissue_) followed a similar trend with time. There was no consistent significant difference between the electrochemical properties of the *in vivo* and *in vitro* electrodes after the implanted period. The differences between the *in vivo* and *in vitro* electrodes during the implanted period show that the evaluation of electrochemical performance of implantable stimulation electrodes cannot be substituted with *in vitro* measurements. After the implanted period, however, the performance of the *in vivo* and *in vitro* electrodes in saline was similar. In addition, the changes observed over time during the post-implantation period regarding the electrochemical properties of the *in vivo* electrodes and R_tissue_ were similar, which indicates that these changes are due to the foreign body response to implantation.

## Introduction

Peripheral nerve stimulation electrodes are currently applied among others to restore vision, hearing, movement, breathing, continence, and to relieve pain (Navarro et al., 2005; Stieglitz and Meyer, 2006; Zhou and Greenbaum, 2009). It is advantageous to make such electrodes as small as possible to minimize tissue trauma, while reducing the risk of activating other excitable tissue (Stieglitz and Meyer, 2006). However, when attempting to achieve a high level of selectivity, the aim to minimize the amount of tissue trauma is often compromised (Navarro et al., 2005). In addition, a small surface area limits transfer of charge, leading to a small volume of tissue that can be excited. This increases the risk of losing the clinical effect due to movement or encapsulation of the electrode, for example. Hence, there is always a trade-off (Merrill, 2009). By carefully choosing the target tissue and meticulously designing the electrode, as well as the implantation procedure and tools (Colvin, 2009), a higher selectivity can be achieved using a less invasive procedure.

The electrode described in this paper will be used to inhibit the bladder muscle. The target tissue for neural stimulation is the dorsal genital nerve (Nakamura and Sakurai, 1984; Dalmose et al., 2003; Fjorback et al., 2006; Martens et al., 2011), which is a sensory branch of the pudendal nerve. The electrode can be implanted using a minimally invasive procedure near the distal end of the nerve. The invasiveness of the procedure is therefore low, while stimulation selectivity is high. Due to the minimally invasive procedure, however, the distance between the electrode and the nerve is not precisely known (range: few mm) and may vary from implantation to implantation. To overcome variation in electrode positioning and orientation, the electrode has a single semi-spherical contact (monopolar), as this provides a rather homogeneous spread of the electrical field in all directions. Furthermore, the electrode's surface area must be sufficiently large to transfer an adequate amount of charge to elicit the desired response without degrading the electrode (Cogan, 2008). For this purpose, the electrode is coated with a 12 μm layer of porous titanium nitride (TiN), which increases the surface area of the electrode (Cunha et al., 2009).

The aims of the current study are (1) to investigate the electrochemical properties of porous TiN electrodes, in terms of impedance spectra and voltage transients, *in vivo* and *in vitro* for a period of 90 days and (2) to compare the electrochemical performance of the electrodes, in terms of their impedance magnitude, voltage transients and charge storage capacity, before and after 90 days. Thirdly, the influence of electrical stimulation, electrochemical impedance spectroscopy (EIS), and voltage transient measurements (VTM) on the performance of the electrodes is investigated by using additional passive electrodes *in vivo* and *in vitro*. This should clarify whether the electrochemical properties of the electrodes are affected by implantation in living tissue, electrical measurements or a combination of these.

## Materials and methods

Fifteen electrodes were coated with a porous TiN film; 10 of these were *in vivo* and five were *in vitro* electrodes. The *in vivo* electrodes were implanted in two Göttingen minipigs. Four of them were used for electrical stimulation and electrochemical measurements (active). These were implanted near the genital nerves of the pigs, one on the left and one on the right side of each animal. The other six were passive electrodes; they were implanted subcutaneously without a wire and were not used for measurements during the implanted period. The *in vitro* electrodes were kept in a container with Ringer solution at 37.5°C for the duration of the study with a distance of 15 cm between the working and counter electrode. Four of the five *in vitro* electrodes were used for measurements (active), while one was passive.

Pre- and post-implantation characterization measurements were made. The surfaces of the electrodes were analyzed using scanning electron microscopy (SEM) and energy dispersive x-ray (EDX). Electrochemical characterization consisted of EIS, VTM, and cyclic voltammetry (CV).

**Figure 1:**
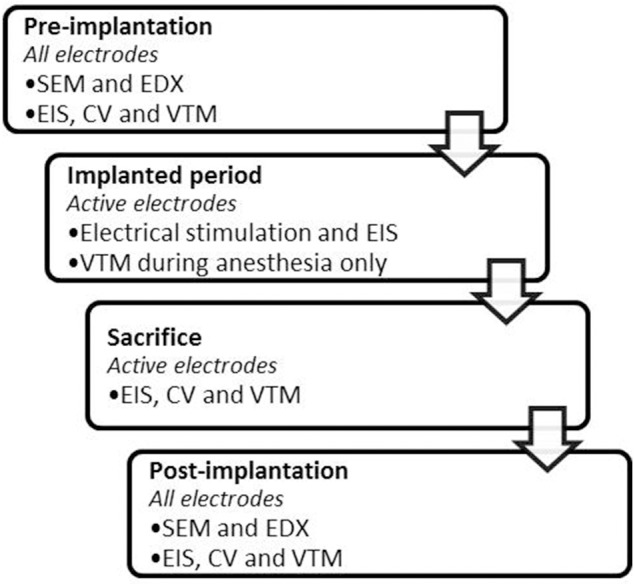
**Timeline of the measurements performed before, during and after the implanted period**.

During the implanted period, the electrode properties of all active electrodes were investigated using electrical stimulation, EIS and VTM. All measurements were performed identically *in vivo* and *in vitro*. At the end of the implanted period, electrochemical characterization measurements were done *in vivo* and *in vitro*. The implanted period was 90 days in order to have sufficient data during the chronic phase. Figure 1 shows the timeline of the different measurements during the study.

### Electrode fabrication

The porous TiN thin films were deposited on the tip of 15 Ti6Al4V substrates (6 mm^2^) and 3 commercially pure Ti-discs (1000 mm^2^) by reactive DC magnetron sputtering using an industrial CemeCon CC800 coating unit. The sputtering chamber was equipped with two magnetrons—each mounted with a 88 × 200 mm Ti target (grade I). A continuous DC power of 2 kW was applied at each magnetron. The depositions were carried out in an Ar/N2 atmosphere, where N2 acts as the reactive gas for the TiN film formation at a total pressure of about 2900 mPa. The Ar/N_2_ gas flow ratio was 180 sccm/240 sccm, resulting in over-stoichiometric TiNx films. The deposition rate was 7.5 nm/min, and the films were synthesized with nominal thickness of 12.5 μm by controlling the deposition time.

**Figure 2:**
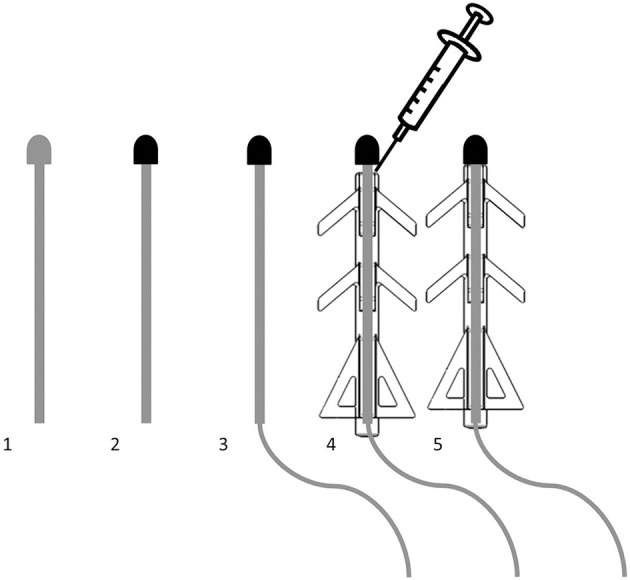
**Fabrication of the electrodes was done in 5 steps, starting with a bare Ti6Al4V pin (1)**. Its tip was sputter coated with porous TiN (2). A 35N LT wire was laser welded to the pin (3). The silicone jacket was slid onto the pin and attached using a silicone adhesive (4). After curing, the electrode was finished (5).

Figure 2 describes the production process of the electrodes. A 35N LT wire, insulated with a thin eTFE coating (Heraeus, Switzerland) was laser welded to the end of the Ti pin and to the Ti disks. The pins used as *in vivo* electrodes were insulated using an injection molded MED-6033 silicone (NuSil, USA) jacket, which was glued to the pin using silicon adhesive MED2-4213 (NuSil, USA). The *in vitro* pins were not insulated, as this would prevent usage of a water tight electrochemical cell.

### Surgical procedure

The electrodes were implanted in two 18 month old Göttingen minipigs. The protocol was approved by the Danish animal experiments inspectorate under license number 2012-15-2934-00177. The animals were first tranquilized and then anesthetized using a bolus injection of propofol. Anesthesia was maintained using intra-venous administration of propofol at 12 ml/h. A concentric needle was inserted into the tissue close to the vagina aiming for the genital nerve while stimulating at 1 Hz. When the threshold for eliciting a contraction of the anal sphincter was less than 5 mA using a 0.2 ms biphasic current pulse, the needle was assumed to be near the genital nerve. The electrodes were then implanted using a Seldinger-like technique. This procedure was performed on the right and left side of the vagina. The active electrodes had a percutaneous exit site at the back of the pigs. The passive electrodes were implanted subcutaneously at various positions on the back and had no percutaneous connection. One counter electrode (TiN coated Ti disk) was implanted at the back of each pig with a percutaneous connection to allow for measurements. The distance between the counter and the working electrodes was approximately 30 cm.

### Electrical measurements

A 3-electrode setup was used for the electrochemical measurements during the pre- and post-implantation measurements, consisting of a TiN working electrode, a platinum foil counter electrode (50 cm^2^) and an Ag|AgCl wire reference electrode (1.6 cm^2^). The *in vitro* measurements were done in an electrochemical cell where the electrodes were in a fixed position to keep the electrolyte resistance constant. During the implanted period, a TiN disk electrode (10 cm^2^) was used to serve as a counter and pseudo-reference electrode both *in vitro* and *in vivo*, which should ideally result in a 0 V open circuit potential. The surface area of the counter electrode was approximately 160 times larger than the surface area of the stimulation electrode to render its influence on the electrochemical measurements negligible.

EIS was used for electrochemical characterization before and after the implanted period, but also for monitoring the tissue response and electrode performance during the implanted period. EIS was performed using Solartron, Model 1294 in conjunction with 1260 Impedance/gain-phase Analyzer (Solartron Analytical, UK) with accompanying SMaRT software installed on a laptop. Table 1 shows the different measurement settings that were used during the pre- and post-implantation characterization measurements and during the implanted period (*in vivo* and *in vitro*). Three different currents were used during the characterization measurements to ensure that the measurement currents were in the linear operation range of the electrode (Ragheb and Geddes, 1990). EIS was performed on every work day during the first weeks after implantation. As the impedance spectra stabilized, the number of measurements per week was reduced. During the measurements, animals were restrained in a custom-built sling.

**Table 1:** **Measurement settings for *in vitro* and *in vivo* EIS measurements**.

	**Pre- and post-implantation**	**Implanted period**
Configuration	3-electrode	2-electrode
Disturbance	Sinusoidal current	Sinusoidal current
Amplitude	1 μA, 5 μA, and 10 μA	10 μA
Frequency range	10 kHz–0.1 Hz	10 kHz–0.1 Hz
Sampling frequency	5 pts/decade	5 pts/decade
Integration time	5 s	2 s

VTM were made using a cathodic-first bipolar symmetric stimulation pulse; each with a phase width of 200 μs. Charge injection limits (Q_inj_) were determined using the maximal cathodic and anodic voltages from which the potential drop due to the electrolyte resistance (IR-drop) was subtracted, E_mc_ and E_ma_, respectively. The IR-drop was calculated by subtracting the potential at 20 μs after pulse cessation from the last data point of the anodic phase (Cogan, 2008). To determine Q_inj_, the current was increased until E_mc_or E_ma_ reached the water window potentials, which were −0.6 V and 0.9 V vs. Ag|AgCl *in vitro* and −0.403 and 1.107 V *in vivo*, as no reference electrode was used. For analysis of the voltage transients, the open-circuit potentials were set to 0 V, this is referred to as normalization. A current of 5 mA was used, as this was expected to be representative for a relatively high stimulation current. *In vivo* VTM and CV were made while the pigs were anesthetized for other purposes (CT scanning and replacing sutures).

CV was performed by cycling the electrode potential between the water window limits, which were -0.6 and 0.9 V vs. Ag|AgCl before and after the implanted period. The *in vitro* limits were determined beforehand using comparable electrodes. The limits used during the implanted period were based on these, though they were shifted to account for not using a reference electrode: −0.403 and 1.107 V. CV was performed at 0.05, 0.1, 0.5, 1.0, 5.0, and 10.0 V/s. The cathodic and anodic charge storage capacity (CSC) of the electrode was defined as the surface area under and over the 0 A axis, respectively. CV and VTM were performed using the VersaSTAT 3 potentio- galvanostat (Princeton applied research, USA).

Electrical stimulation was performed weekly using a Teca Synergy T-EP—EMG/EP Monitoring System (Oxford Instruments Medical, UK). The pigs were restrained using a custom built sling while stimulation was performed. The lowest current at which an anal sphincter contraction was observed visually, using a pulse train of 5 0.2 ms biphasic current pulses, was considered the threshold. Stimulation was increased to investigate whether the contractions increased in strength until side-effects occurred, such as contractions of other muscles.

### Analytical measurements

SEM (Nova 600, FEI Company, Netherlands) images of all electrodes were recorded at 150, 900, and 2500x magnification before and after the implanted period to determine whether any changes occurred on the electrodes' surface structure due to the experiments. EDX (EDAX, AMETEK, Germany) spectra were made of all electrodes before and after the implanted period to determine whether there were any changes in surface chemistry of the electrodes.

Inductively coupled plasma atomic emission spectroscopy (ICP-AES) (AMETEK Spectro Arcos, AMETEK, Germany) was used for the determination of trace elements in the Ringer solution in which the *in vitro* electrodes were kept. The total volume of the Ringer solution (1.2 l) was condensed to a volume of 60 ml, which was used for ICP-AES.

After the animals were euthanized, tissue samples containing the *in vivo* electrodes were removed and drop fixed in formalin. One active electrode, one passive electrode and the subcutaneous tissue adjacent to the counter electrode was taken from each animal. When fixed, the tissue samples were sliced and stained using hematoxylin and eosin (H&E) for histological investigation.

### Statistical analysis

The pre- and post-implantation data were statistically analyzed using SPSS. The data entered into SPSS were the cathodic CSC at 0.05 V/s, the impedance magnitude (|Z|) at 0.1 Hz, and E_mc_ of the VTM using a current of 20 mA. The data were entered into a linear mixed model with a repeated variable (time). The dependent variables were the measurement values of each of the different measurements and the fixed factors of the mixed model were time (pre- or post), type (*in vitro* or *in vivo*) and usage (active or passive). Significance tests were performed using pairwise comparisons with Bonferroni's adjustment for multiple comparisons. Estimated marginal means (EMM) and standard errors (SE) were obtained to compare the single and combined influences of the fixed factors, while keeping the other factors of the model constant.

## Results

Due to broken wires, it was impossible to complete the measurements of some electrodes. Two welds of the active *in vivo* electrodes broke on day 4 and day 28 after implantation. The results of these electrodes were used until the welds broke. The weld of the counter electrode of pig 2 (electrode 3 and 4) broke on day 80 and there is thus no data from electrode 3 between day 80 and 90. *In vivo* measurements on electrode 3 in pig 2 on the day of sacrifice (day 90) was done by surgically exposing the counter electrode. Although the broken wires were unfortunate and it limits the amount the data obtained in this study, sufficient longitudinal (over 2 months) data was obtained for 2 electrodes during the chronic phase after implantation.

### Implanted period

**Figure 3:**
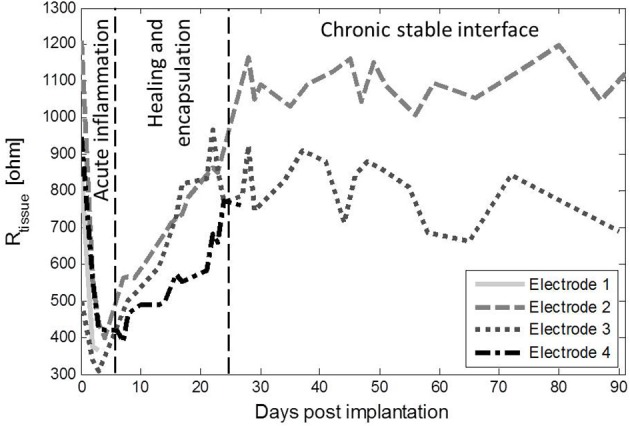
**R_tissue_ of the 4 active ***in vivo*** electrodes as a function of time after implantation**. There was a drop of the impedance during the first days after implantation, corresponding to the acute inflammation phase (Grill and Mortimer, 1994; Williams et al., 2007). R_tissue_ increased, which corresponded to healing of the tissue and encapsulation of the electrode (Grill and Mortimer, 1994; Williams et al., 2007; Lempka et al., 2009), after which R_tissue_ stabilized.

**Figure 4:**
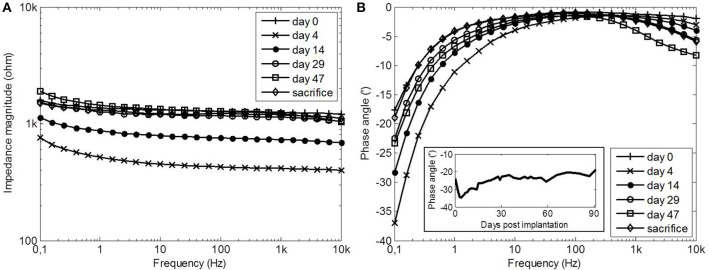
**Impedance magnitude (A) and phase angle (B) spectra of electrode 2 at selected time points after implantation**. The insert shows the average phase angle of all electrodes at 0.1 Hz as a function of time.

Figure 3 shows the impedance magnitude at 10 kHz, which is representative of the tissue impedance (R_tissue_), as the phase angle approached 0 from frequencies higher than 10 Hz (Figure 4). R_tissue_ was relatively high on day 0, because bodily fluids may not have completely surrounded and filled the pores of the electrode. The impedance then dropped due to accumulation of fluids around the electrode (Grill and Mortimer, 1994; Williams et al., 2007), after which the tissue impedance increased due to the formation of a fibrous capsule around the electrode (Grill and Mortimer, 1994; Williams et al., 2007; Lempka et al., 2009). The increasing trend was followed by stabilization of the tissue impedance. The electrolyte impedance, measured using the *in vitro* electrodes, remained constant throughout the study at 226 Ω.

The impedance magnitude and phase angle spectra at selected time points are shown for electrode 2 in Figure 4. The impedance magnitude spectrum (Figure 4A) was lowest on day 4, after which it increased and stabilized at the level of day 0. The phase angle spectra (Figure 4B) showed a similar trend at the frequencies below 10 Hz (see insert). The phase angle was lowest on day 4, after which it increased to the level of day 0. The phase angle was close to zero between 10 Hz and 1 kHz during the entire implanted period. At frequencies above 1 kHz, the phase angle decreased with time. During the entire implanted period, none of the properties of the *in vitro* electrodes changed.

**Figure 5:**
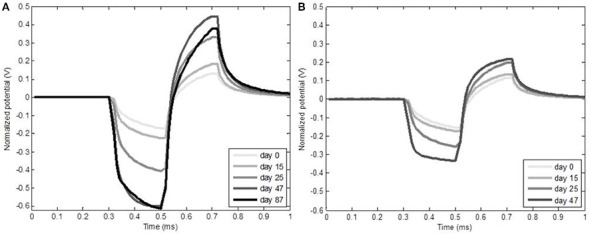
**Normalized voltage transients of ***in vivo*** electrode 2 (A) and 3 (B) increased with time**. The anodic voltage transients appeared to stop increasing from day 25. The cathodic voltage transient of electrode 2 stopped to increase from day 47. No *in vivo* voltage transients could be measured for electrode 3 on day 87, as the wire of the counter electrode was missing.

**Figure 6:**
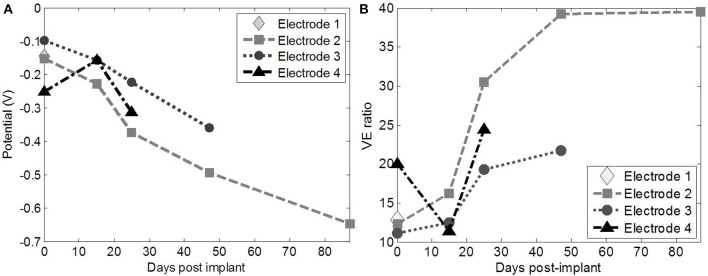
**The ratio of the ***in vivo***/***in vitro*** voltage excursions (VE ratio) (A) and E_mc_ (B) for each of active ***in vivo*** electrodes the electrodes**. Especially electrode 2 and 3 were similarly but to a different extent affected during the implanted period.

Figure 5 shows that the normalized voltage excursions of the *in vivo* electrodes increased with time. The increasing trend in the anodic phase of the voltage transients leveled off from day 25. For the cathodic phase the voltage transients on day 47 and day 87 had the same magnitude. No increasing trend was observed for *in vitro* electrodes, which had an average cathodic and anodic voltage excursion of 14 ± 2 mV and 6.5 ± 1 mV, respectively, throughout the implanted period. The average voltage excursion of the *in vivo* electrodes on day 0 was 13 times larger than the average voltage excursion of the *in vitro* electrodes. Figure 6A shows that the voltage transients of each of the electrodes were differently affected during the implanted period. The increase in the voltage excursions leveled off for electrodes 2 and 3, which had voltage excursions that were 39 and 21 times larger than those of the *in vitro* electrodes, respectively.

E_mc_ of the individual electrodes decreased with time (Figure 6B), due to the influence of the OCP, and the water window limit (−0.403 V) was exceeded on day 47 and 87 for electrode 2. Q_inj_ was measured using electrode 2 and 3 *in vivo* after 90 days; these were 7 and 20 μC/cm^2^, respectively.

**Figure 7:**
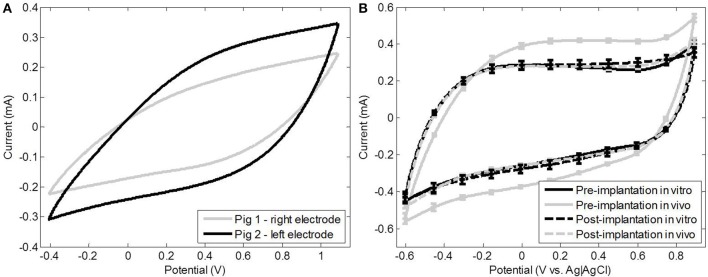
**The ***in vivo*** CV of electrode 2 and 3 at sacrifice shows a more resistive than capacitive behavior (A)**. Average CV of the *in vivo* and *in vitro* electrodes pre- and post-implantation showing the capacitive behavior that is characteristic for TiN **(B)**.

Figure 7A shows the CV of two implanted electrodes just before sacrifice, which was more diagonal than during *in vitro* measurements (Figure 7B), due to the encapsulation tissue around the electrode contact (Fjorback et al., 2006). The CSC of both *in vivo* electrodes was lower (98.2 and 64.8 mC/cm^2^) than the CSC of the *in vitro* electrodes (138.9 ± 6 mC/cm^2^) characterized at the end of the implanted period. The CSC of the *in vivo* electrodes was at the pre-implantation level when measured *in vitro* after the implanted period.

It was possible to consistently elicit a reflex contraction of the anal sphincter using electrode 2. During the first 3 weeks the threshold for eliciting a reflex was 2.5 mA, after which the threshold decreased to 1.5 mA. In pig 2 (electrodes 3 and 4), a contraction of the anal sphincter due to stimulation could not be distinguished, as rhythmic sphincter contractions also occurred without stimulation.

### Pre- and post-implanted period

The electrodes were divided into 4 groups: active *in vitro*, passive *in vitro*, active *in vivo*, and passive *in vivo*. Measurements were made before and after the implanted period and comparisons were made before and after the implanted period between the different groups and between the pre- and post-implantation measurements within the different groups.

Before the implanted period, the *in vitro* electrodes had a lower |Z|, higher CSC and lower voltage excursions than the *in vivo* electrodes (*p* < < 0.05 for all measurements). The only significant difference after the implanted period was a lower |Z|of the active *in vitro* electrodes than the active *in vivo* electrodes (*p* = 0.02).

All electrode groups had a higher |Z|after as compared to before the implanted period (*p* < < 0.05) (Table 2). The post-implantation voltage excursions of the active *in vitro* electrodes had increased significantly (*p* = 0.007) as compared to before the implanted period; E_mc_ decreased accordingly from −47 ± 7 to −72 ± 5 mV (Table 3). The voltage excursions of the passive *in vivo* electrodes, on the other hand, had decreased significantly (*p* = 0.009) after as compared to before the implanted period; E_mc_ increased accordingly from −1.3±6 to −83 ± 4 mV (Table 3). There were no significant differences between the voltage excursions in the pre- and post-implantation measurements of the passive *in vitro* electrodes and the active *in vivo* electrodes. Furthermore, the *in vitro* electrodes had a significantly higher CSC before the implanted period, as compared to after (*p* < < 0.05) (Table 4). The surface area of the voltammogram of the *in vitro* electrodes was smaller after the implanted period as compared to before, but showed no qualitative changes (**Figure 9B**). The voltammogram of the *in vivo* electrodes did not show any differences before as compared to after the implanted period (**Figure 9B**). The CV of all electrodes, before and after implantation, became diagonal at a sweep rate of 0.5 V/s, confirming the high level of porosity of the electrodes (Cunha et al., 2009).

**Table 2:** **Mean |Z| at 0.1 Hz of the different groups of electrodes before and after the implanted period (EMM ± SE)**.

**Electrode group**	**Pre-implantation (Ω)**	**Post-implantation (Ω)**
Passive in vitro^*^	359 ± 19	807 ± 32
Active in vitro^*^	349 ± 10	828 ± 16
Passive in vivo^*^	499 ± 8	801 ± 13
Active in vivo^*^	526 ± 10	887 ± 16

* *Significant difference between pre- and post-implantation (p < 0.05)*.

**Table 3:** **Mean E_mc_ of the different groups of electrodes before and after the implanted period using a 20 mA current (EMM ± SE)**.

**Electrode group**	**Pre-implantation (mV)**	**Post-implantation (mV)**
Passive *in vitro*	−51±14	−65± 11
Active in vitro^*^	−47±7	−72± 5
Passive in vivo^*^	−1.3±6	−83± 4
Active *in vivo*	−82±7	−93± 5

* *Significant difference between pre- and post-implantation (p < 0.05)*.

**Table 4:** **Mean CSC_C_ of the different groups of electrodes before and after the implanted period using a sweep rate of 0.05 V/s (EMM ± SE)**.

**Electrode group**	**Pre-implantation**	**Post-implantation**
	**(mC/cm^2^)**	**(mC/cm^2^)**
Passive in vitro^*^	165 ± 4	109 ± 6
Active in vitro^*^	173 ± 2	116 ± 3
Passive *in vivo*	124 ± 2	127 ± 3
Active *in vivo*	121 ± 2	121 ± 3

* *Significant difference between pre- and post-implantation (p < 0.05)*.

**Figure 8:**
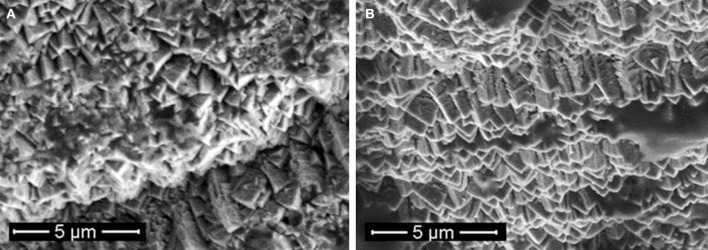
**SEM of an active ***in vivo*** electrode before (A) and after (B) the implanted period**.

**Figure 9:**
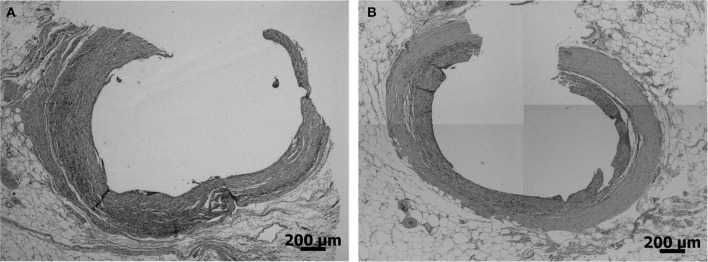
**Histology of and active (A) and a passive (B) ***in vivo*** electrode**.

The SEM images showed no cracks or pits in the bulk electrode area and displayed an open columnar microstructure (Figure 8). However, other particles were found on the electrodes. EDX revealed that these were carbon particles and they accounted for an average of 21 wt-% (range: 5–60 wt-%). The particles could not be. removed using isopropyl alcohol, ultrasonic or electrochemical treatment. The electrodes were cleaned and sterilized before implantation and no signs of infection were found in the animals, although histology at the end of the study revealed the presence of eosinephilic granulocytes in the fibrous tissue capsule (Figure 9). SEM after explantation showed no changes in the surface structure (Figure 8), although EDX showed an increase in the amount of carbon to an average of 30 wt-%. This was due to the presence of (fixated) cells on the *in vivo* electrodes and remnants of Ringer salts in the pores of the *in vitro* electrodes. The levels of oxygen before and after the implanted period were the similar. Low levels of oxygen are typically found on TiN surfaces, as a small oxide layer is formed due to air exposure (Norlin et al., 2005; Avasarala and Haldar, 2010). No electrode particles were found in the tissue surrounding the electrodes. Neither were any TiN or silicone traces found in the ICP characterization of the Ringer solution in which the *in vitro* electrodes were kept during the 90 days implanted period.

## Discussion

The contamination of the electrodes with carbon particles was unfortunate, but as the electrodes performed outstanding electrochemically (Weiland et al., 2002) with an electrochemical surface area of about 23 mm^2^ (384 times the geometrical surface area), it was decided to test the electrodes *in vivo* and *in vitro* after autoclaving them. No signs of infection were observed and the healing period compares very well to the duration found by Grill and Mortimer (1994). No carbon particles were found in the tissue and particles were still found on the electrodes after explantation. In addition, the particles covered only approximately 2% of the entire surface area of the electrodes. The extent to which the presence of these particles may have influenced the electrochemical measurements is therefore limited.

### Electrode properties *in vivo*

Three interesting changes were observed during the *in vivo* measurements: (1) the large voltage excursions *in vivo* as compared to *in vitro*, (2) the increasing trend of the normalized voltage excursions *in vivo*, and (3) the similarity between the trends in the phase angle below 10 Hz and R_tissue_. To our knowledge none of these observations have been described for TiN electrodes before.

The similar trend in R_tissue_ and the phase angle below 10 Hz was partly caused by the change in R_tissue_. When a simplified Randles model (a parallel RC circuit and a series resistor) is assumed, the EIS spectrum (Figure 4A) displays the behavior of the capacitor and the series resistor. When the resistance of the series resistor increases, the frequency at which the capacitive behavior is visible in the spectrum decreases. This is what happens as R_tissue_ increases and the insert in Figure 4B shows that the average phase angle at 0.1 Hz indeed shows the same trend as R_tissue_. This, however, could not explain the observed trend completely. We therefore investigated the double layer capacitance (C_dl_) using the simplified Randles model. C_dl_ was derived from the data as follows (Wei and Grill, 2009):

$$C_{dl}\left(\omega \right)=-\frac{Z^{\prime \prime }}{\omega ({\left(Z^{\prime }-R_{\text{tissue}}\right)}^{2}+{Z^{\prime \prime }}^{2})}$$

**Figure 10:**
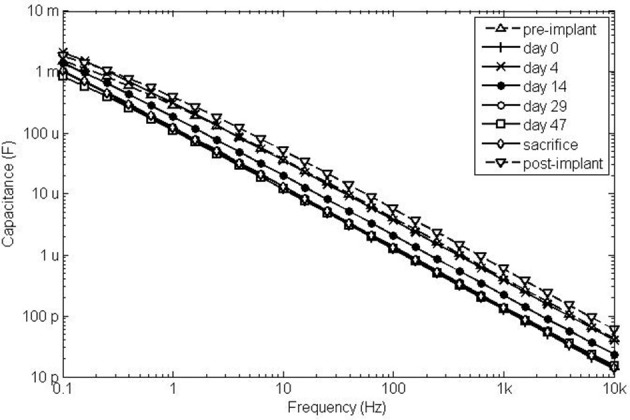
**C_dl_ as a function of frequency shows a very similar trends as R_tissue_**. On day 0, C_dl_ was relatively low, whee R_tissue_ was relatively high. C_dl_ was increased on day 4, where R_tissue_ was decreased and C_dl_ decreased to day-0 value at which it remained from day 29, while R_tissue_ increased and stabilized during the same time course.

Where ω is the angular frequency, *Z*′ is the real part of the impedance and *Z*″ is the imaginary part of the impedance. Figure 10 shows that *C*_dl_ follows the same trend as *R*_tissue_, which was consistent over the entire frequency range. *C*_dl_ is on the same level as *C*_*dl*_ of the *in vitro* electrodes on day 4. The same trend was found when the data was fitted to the simplified Randles model, signifying that the total value of *C*_dl_ is affected by changes in the medium in which the electrode is immersed. A decreasing trend in electrode capacitance was also found for a deep brain stimulation electrode, which was related to the accumulation of cells around the electrode, since no changes of the electrode impedance in albumin solution were observed (Lempka et al., 2009). We suspect that this is the same for porous TiN, as both proteins and cells have been reported to adhere well to a TiN surface (Groessner-Schreiber et al., 2002). This also explains why C_dl_ was at the pre-implantation level after the implanted period. We have investigated smooth TiN electrodes *in vivo* before, where a decreasing trend in C_dl_ was not observed (Meijs et al., 2012). This may indicate that this trend is specific for porous TiN electrodes, as both surface chemistry and topography influence cell adhesion (Cyster et al., 2003).

The *in vivo* voltage excursions were 13 times larger than those measured *in vitro*, which corresponds well to the difference in pulsing capacitance *in vivo* compared to *in vitro*. Such differences have been described for platinum micro and macro stimulation electrodes (Musa et al., 2009; Wei and Grill, 2009) and is likely due to the adsorption of proteins (Musa et al., 2009), which occurs before cells adsorb to the electrode surface (Groessner-Schreiber et al., 2002; Lempka et al., 2009). These proteins pose a diffusion limitation for the counter ions *in vivo* as compared to Ringer solution, which has a purely ionic content (Cogan, 2006, 2008; Lempka et al., 2009). As cells adhere to the surface the diffusion limitation increases further, causing increased impedance, IR drop and voltage excursions (Newbold et al., 2004; Gimsa et al., 2005).

The only other study to investigate charge injection of chronically implanted neural stimulation electrodes is by Kane et al. (2011, 2013). Their results (Kane et al., 2013) show decreasing values of E_mc_ over time, which is in accordance with our findings. One major methodological difference between the study by Kane et al. (2011) and this study is the controlling of the inter-pulse potential. Therefore, E_mc_ was not influenced by changes in the open circuit potential in their results. We attempted to achieve an open circuit potential of approximately 0 V by implanting a pseudo-reference electrode of the same material as the working electrode. The open circuit potentials deviated typically less than 40 mV from 0 V. Kane et al. (2011) used CV and EIS *in vivo* and attempted to determine the cause of decreasing E_mc_. This was inconclusive due to leakage issues (Kane et al., 2011, 2013). In the current study CV was only performed at sacrifice, where the CSC of the *in vivo* electrodes was 47 and 71% of their *in vitro* CSC before implantation. This ratio decreased with sweep rate and is likely due to the diffusion limitation posed by the tissue (Cogan, 2008; Kane et al., 2013), which is confirmed by the fact that the *in vivo* electrodes did not have a lower CSC post-implantation as compared to pre-implantation. The difference in CSC at the lowest sweep rate was similar to the difference found between the *in vivo* and *in vitro* values of *C*_dl_. It must be noted, however, the safe potential limits were not determined *in vivo*, instead the limits used for *in vitro* measurement were applied *in vivo*. The width of the safe potential window is therefore the same *in vivo* and *in vitro*, but it is possible that the window used *in vivo* was too conservative (see Figure 9).

### Pre- and post-implanted period

None of the aforementioned studies have electrochemically investigated the electrodes *in vitro* before and after implantation and to our knowledge this is the first study to do so. The purpose of these investigations was to determine the cause of any degradation of the electrochemical electrode properties by comparing active *in vitro*, passive *in vitro*, active *in vivo*, and passive *in vivo* electrodes. As the statistical power of this study is low, we chose to discuss only the results that were consistent for all electrodes across all measurements. With consistent is meant that a decrease in CSC was accompanied by the expected decrease in E_mc_and increase in |Z|. These are: (1) Before implantation the *in vitro* electrodes had a larger CSC, a larger E_mc_ and lower |Z|than the *in vivo* electrodes. (2) After the implanted period, the *in vitro* electrodes had a smaller CSC, a smaller E_mc_and a higher |Z|than before the implanted period.

We suggest the following three theories to explain these results:

- The *in vitro* environment is more corrosive than the *in vivo* environment. This theory is supported by data from Hibbert et al. (2001) and Roblee et al. (1980). It implies that the electrochemical properties of the *in vitro* electrodes deteriorated during the implanted period, which is not supported by the data recorded during the implanted period. Nevertheless, the CV performed at the end of the implanted period could also have deteriorated the electrochemical properties of the *in vitro* electrodes (Norlin et al., 2005).
- Heterogeneity in the active *in vivo* electrode group. As the connection to some of the electrodes was lost quite early in the study, the group of active *in vivo* electrodes is rather heterogeneous. Despite the heterogeneity, the standard errors in the group of active *in vivo* electrodes are no different from the other electrode groups.
- Silicone adhesive had covered the edges of *in vivo* electrodes before the implanted period and this was removed when removing the silicone jackets. It is assumed that the electrical contacts of all electrode pins were the same before fabrication. This seems logical, as all uncoated pins came from the same batch, all pins were coated at the same time and the *in vitro* and *in vivo* electrodes were randomly chosen. It implies that all electrodes deteriorated equally during the implanted period, as there were no consistent significant differences between any of the electrode groups after the implanted period. The deterioration of the *in vivo* electrodes is then assumed to be concealed by the increased surface area due to removal of the silicone adhesive.

The actual explantation is likely a combination of these theories, as the first 2 fail to explain the difference between the *in vitro* and *in vivo* electrodes and the last does not give any cause for the deterioration of the electrochemical properties of the electrodes.

### Implications of the study

It is obvious from this study, that *in vitro* and *in vivo* charge injection properties cannot be compared. Attempts have been made to determine which electrolyte compares best to the *in vivo* situation (Cogan, 2006; Cogan et al., 2007). These studies show that the choice of electrolyte is very important, but also that the best performing electrolyte still shows significant shortcomings as compared to the *in vivo* data (Cogan, 2006). Comparing the electrolyte resistance to R_tissue_, we find that the electrolyte resistance (226 Ω) compares well to R_tissue_ at its lowest level (300–500 Ω on day 2–8) with approximately double the distance between the working and counter electrode *in vivo*. During the acute inflammatory phase, the electrode is surrounded by bodily fluids (Grill and Mortimer, 1994; Williams et al., 2007), But as a dense capsule is formed around the electrodes (Grill and Mortimer, 1994; Williams et al., 2007; Lempka et al., 2009), even the electrolyte resistance does not resemble that of the tissue. This has been reported to influence some of the electrochemical properties of iridium oxide electrode; EIS in terms of R_tissue_ and the voltage transients in terms of IR drop and voltage excursions (Cogan et al., 2007). As iridium oxide electrodes inject charge via capacitive and faradic pathways (Cogan, 2006; Cogan et al., 2007), this may be different for TiN employs only capacitive pathways within the safe potential limits. We have therefore conducted some experiments with different concentrations of Ringer solution (1/4, 1/6, and 1/12-strength) and found no dependence of the voltage excursions on concentration. Adding proteins to the solution may also deteriorate the electrochemical properties of stimulation electrodes (Cyster et al., 2003; Farcas et al., 2011; Kane et al., 2011), but in the case of platinum it has a protective effect against dissolution as well (Roblee et al., 1980; Hibbert et al., 2001). The first part seems to also hold for TiN electrodes, as the impedance magnitudes and voltage transients were higher directly after implantation than before, despite the similar conductivity of the tissue and Ringer solution in the acute inflammation phase. Proteins may also have a protective effect for TiN electrodes, as most of the changes observed *in vivo* were reversed after explantation.

The main advantage of the electrode investigated in this study is that it can be implanted using a minimally invasive procedure, with as a drawback that this requires higher charge injection limit than with an electrode that is tied around the nerve, for example. But with the increasing voltage excursions, the question arises whether these electrodes are still good enough. The electrochemical surface area of the electrodes is 384 times larger than the surface area of a smooth electrode with the same geometrical surface area measured in Ringer solution. At the end of the implanted period, CV was performed *in vivo* and the CSC was decreased to 47–71% compared to their *in vitro* CSC. The voltage excursions, however, were more than 10 times larger *in vivo* compared to *in vitro*, which corresponded to a loss of surface area under pulsing conditions of about 93%. The voltage transient of a smooth TiN electrode in Ringer was only 4 times larger than the voltage transients of the implanted electrodes. This indicates that the electrochemical surface area of the porous electrode available under *in vivo* pulsing conditions is only 4 times larger than the geometrical surface area. As the voltage transients continued to increase, however, this ratio deteriorated to 1.2 for electrode 2 and thus making its performance *in vivo* comparable to that of smooth TiN in Ringer solution. To make a fair comparison, however, smooth and porous TiN should be tested *in vivo* to determine to which extent and during which phase (acute, encapsulation, or chronic) the porous electrodes are affected more than the smooth electrodes by a diffusion limitation. Based on this information, the surface structure and porosity of the coating could be tailored to increase the *in vivo* performance. This is necessary, as the Q_inj_ found at the endpoint of the implanted period allows for injection of 2–6 mA, which is not enough to obtain the clinical effect in humans (Nakamura and Sakurai, 1984; Dalmose et al., 2003; Fjorback et al., 2006; Martens et al., 2011).

Additional work could also be done to better understand the difference between *in vivo* and *in vitro* voltage transients. *In vivo* VTM and CV could be performed more frequently during the implanted period to obtain a better understanding of the changes that occur during the implanted period and to investigate whether there is a relationship between the changing electrochemical properties of the electrodes and the encapsulation process.

## Funding

This research was funded by the Danish National Advanced Technology Foundation.

### Conflict of interest statement

The authors declare that the research was conducted in the absence of any commercial or financial relationships that could be construed as a potential conflict of interest.

## References

[B1] Avasarala B. Haldar P. (2010). Electrochemical oxidation behavior of titanium nitride based electrocatalysts under PEM fuel cell conditions. Electrochim. Acta 55, 9024–9034. 10.1016/j.electacta.2010.08.035

[B2] Cogan S. F. (2006). *In vivo* and *in vitro* differences in the charge-injection and electrochemical properties of iridium oxide electrodes. Annu. Int. Conf. IEEE Eng. Med. Biol. Soc. 2006, 882–885. 10.1109/IEMBS.2006.259654 17946868

[B3] Cogan S. F. (2008). Neural stimulation and recording electrodes. Annu. Rev. Biomed. Eng. 10, 275–309. 10.1146/annurev.bioeng.10.061807.160518 18429704

[B4] Cogan S. F. Troyk P. R. Ehrlich J. Gasbarro C. M. Plante T. D. (2007). The influence of electrolyte composition on the *in vitro* charge-injection limits of activated iridium oxide (AIROF) stimulation electrodes. J. Neural. Eng. 4, 79–86. 10.1088/1741-2560/4/2/008 17409482

[B5] Colvin M. (2009). An effective design process for the successful development of medical devices, in Implantable Neural Prostheses 2, eds Zhou D. Greenbaum E. (New York, NY: Springer-Verlag), 345–359.

[B6] Cunha L. T. Pedrosa P. Tavares C. J. Alves E. Vaz F. Fonseca C. (2009). The role of composition, morphology and crystalline structure in the electrochemical behaviour of TiNx thin films for dry electrode sensor materials. Electrochem. Acta 55, 59–67. 10.1016/j.electacta.2009.08.004

[B7] Cyster L. A, Parker, K. G. Parker T. L. Grant D. M. (2003). The effect of surface chemistry and nanotopography of titanium nitride (TiN) films on 3T3-L1 fibroblasts. J. Biomed. Mater. Res. A 67, 138–147. 10.1002/jbm.a.10087 14517871

[B8] Dalmose A. L. Rijkhoff N. J. M. Kirkeby H. J. Nohr M. Sinkjaer T. Djurhuus J. C. (2003). Conditional stimulation of the dorsal penile/clitoral nerve may increase cystometric capacity in patients with spinal cord injury. Neurourol. Urodyn. 22, 130–137. 10.1002/nau.10031 12579630

[B9] Farcas M. Cosman N. P, Ting, D. K. Roscoe S. G. Omanovic S. (2011). A comparative study of electrochemical techniques in investigating the adsorption behaviour of fibrinogen on platinum. J. Electroanal. Chem. 649, 206–218. 10.1016/j.jelechem.2010.04.004

[B10] Fjorback M. V. Rijkhoff N. Petersen T. Nohr M. Sinkjaer T. (2006). Event driven electrical stimulation of the dorsal penile/clitoral nerve for the management of neurogenic detrusor overactivity in multiple sclerosis. Neurourol. Urodyn. 25, 349–355. 10.1002/nau.20170 16673380

[B11] Gimsa J. Habel B. Schreiber U. van Rienen U. Strauss U. Gimsa U. (2005). Choosing electrodes for deep brain stimulation experiments–electrochemical considerations. J. Neurosci. Methods 142, 251–265. 10.1016/j.jneumeth.2004.09.001 15698665

[B12] Grill W. M. Mortimer J. T. (1994). Electrical properties of implant encapsulation tissue. Ann. Biomed. Eng. 22, 23–33. 10.1007/BF02368219 8060024

[B13] Groessner-Schreiber B. Neubert A. Müeller W. D. Hopp M. Griepentrog M. Lange K. (2002). Fibroblast growth on surface-modified dental implants: an *in vitro* study. J. Biomed. Mater. Res. A 64, 591–599. 10.1002/jbm.a.10417 12601769

[B14] Hibbert D. B. Weitzer K. Carter P. (2001). Voltammetry of platinum in artificial perilymph solution. J. Electrochem. Soc. 148, E1–E7. 10.1149/1.1344543

[B15] Kane S. R. Cogan S. F. Ehrlich J. Plante T. D. McCreery D. B. (2011). Electrical performance of penetrating microelectrodes chronically implanted in cat cortex. Annu. Int. Conf. IEEE Eng. Med. Biol. Soc. 2011, 5416–5419. 10.1109/iembs.2011.6091339 22255562 PMC7439252

[B16] Kane S. R. Cogan S. F. Ehrlich J. Plante T. D. McCreery D. B. Troyk P. R. (2013). Electrical performance of penetrating microelectrodes chronically implanted in cat cortex. IEEE Trans. Biomed. Eng. 60, 2153–2160. 10.1109/TBME.2013.2248152 23475329 PMC7441534

[B17] Lempka S. F. Miocinovic S. Johnson M. D. Vitek J. L. McIntyre C. C. (2009). *In vivo* impedance spectroscopy of deep brain stimulation electrodes. J. Neural Eng. 6, 1–11. 10.1088/1741-2560/6/4/046001 19494421 PMC2861504

[B18] Martens F. M. J. Heesakkers J. P. F. A. Rijkhoff N. J. M. (2011). Minimal invasive electrode implantation for conditional stimulation of the dorsal genital nerve in neurogenic detrusor overactivity. Spinal Cord 49, 566–572. 10.1038/sc.2010.134 20921957

[B19] Meijs S. Fjorback M. Rijkhoff N. J. M. (2012). Chronic electrochemical investigation of titanium nitride stimulation electrodes *in vivo* in Converging clinical and engineering research on neurorehabilitation, eds Pons J. L. Torricelli D. Pajaro M. (Heidelberg: Springer-Verlag), 421–425.

[B20] Merrill D. R. (2009). The electrochemistry of charge injection at the electrode/tissue interface, in Implantable Neural Prostheses 2, eds Zhou D. Greenbaum E. (New York, NY: Springer-Verlag), 85–138.

[B21] Musa S. Welkenhuysen M. Prodanov D. Eberle W. Bartic B. Nuttin B.. (2009). *In vitro* and *in vivo* electrochemical characterization of a microfabricated neural probe. Annu. Int. Conf. IEEE Eng. Med. Biol. Soc. 2009, 7143–7146. 10.1109/iembs.2009.5335362 19965265

[B22] Nakamura M. Sakurai T. (1984). Bladder inhibition by penile electrical stimulation. Br. J. Urol. 56, 423–415. 10.1111/j.1464-410X.1984.tb05833.x 6335974

[B23] Navarro X. Krueger T. B. Lago N. Micera S. Stieglitz T. Dario P. (2005). A critical review of interfaces with the peripheral nervous system for the control of neuroprostheses and hybrid bionic systems. J. Peripher. Nerv. Syst. 10, 229–258. 10.1111/j.1085-9489.2005.10303.x 16221284

[B24] Newbold C. Richardson R. Huang C. Q. Milojevic D. Cowan R. Shepherd R. (2004). An *in vitro* model for investigating impedance changes with cell growth and electrical stimulation: implications for cochlear implants. J. Neural Eng. 1, 218–227. 10.1088/1741-2560/1/4/005 15876642

[B25] Norlin A. Pan J. Leygraf C. (2005). Investigation of electrochemical behavior for stimulation/sensing materials for pacemaker electrode applications: Pt, Ti, and TiN coated electrodes. J. Electrochem. Soc. 152, J7–J15. 10.1149/1.1842092

[B26] Ragheb T. Geddes L. A. (1990). Electrical properties of metallic electrodes. Med. Biol. Eng. Comput. 28, 182–186. 10.1007/BF02441775 2376994

[B27] Roblee L. S, McHardy, J. Marston J. M. Brummer S. B. (1980). Electrical stimulation with Pt electrodes V. The effect of protein on Pt dissolution. Biomaterials 1, 135–139. 10.1016/0142-9612(80)90035-6 7470564

[B28] Stieglitz T. Meyer J.-U. (2006). Neural implants in clinical practice, in BioMEMS, ed Urban G. A. (Dordrecht: Springer-Verlag), 41–70.

[B29] Wei X. F. Grill W. M. (2009). Impedance characteristics of deep brain stimulation electrodes *in vitro* and *in vivo*. J. Neural Eng. 6, 1–9. 10.1088/1741-2560/6/4/046008 19587394 PMC3066196

[B30] Weiland J. D, Anderson, D. J. Humayun M. S. (2002). *In vitro* electrical properties for iridium oxide versus titanium nitride stimulating electrodes. IEEE Trans. Biomed. Eng. 49, 1574–1579. 10.1109/TBME.2002.805487 12549739

[B31] Williams J. C. Hippensteel J. A. Dilgen J. Shain W. Kipke D. R. (2007). Complex impedance spectroscopy for monitoring tissue responses to inserted neural implants. J. Neural Eng. 4, 410–423. 10.1088/1741-2560/4/4/007 18057508

[B32] Zhou D. Greenbaum E. (2009). Implantable Neural Prostheses I Devices and Applications. New York, NY: Springer-Verlag.

